# Renal Effects of Dental Amalgam in Children: The New England Children’s Amalgam Trial

**DOI:** 10.1289/ehp.10504

**Published:** 2007-11-23

**Authors:** Lars Barregard, Felicia Trachtenberg, Sonja McKinlay

**Affiliations:** 1 Department of Occupational and Environmental Medicine, Sahlgrenska University Hospital and Academy, Gothenburg, Sweden; 2 New England Research Institutes, Watertown, Massachusetts, USA

**Keywords:** albumin, alpha-1-microglobulin, children, dental amalgam, γ-glutamyl transpeptidase, glomerular kidney function, NAG, renal function, tubular kidney function

## Abstract

**Background:**

Mercury is nephrotoxic and dental amalgam is a source of mercury exposure.

**Methods:**

Children 6–10 years of age (*n* = 534) with two or more posterior teeth with caries but no prior amalgam restorations, were randomized to one of two treatments—amalgam or resin composite (white fillings)—used for caries treatment during 5 years of follow-up. The primary outcome was change in IQ, but important secondary outcomes were effects on markers of glomerular and tubular kidney function: urinary excretion of albumin, alpha-1-microglobulin (A1M), γ-glutamyl transpeptidase (γ-GT), and *N-*acetyl-β-d-glucosaminidase (NAG). These markers were measured on several occasions during the trial, together with urinary mercury and covariates. We evaluated the results using repeated-measures analyses.

**Results:**

There were no significant differences between treatment groups in average levels of renal biomarkers, nor significant effects of number of dental amalgams on these markers. There was, however, a significantly increased prevalence of microalbuminuria (MA) among children in the amalgam group in years 3–5 (adjusted odds ratio 1.8; 95% confidence interval, 1.1–2.9). Most of these cases are likely to be temporary MA, but 10 children in the amalgam group had MA in both years 3 and 5, versus 2 children in the composite group (*p* = 0.04). There were no differences in the occurrence of high levels of renal tubular markers (A1M, γ-GT, or NAG).

**Conclusions:**

The increase in MA may be a random finding, but should be tested further. The results did not support recent findings in an observational study of an effect of low-level mercury on tubular biomarkers in children.

Mercury is a toxic heavy metal occurring in several physical and chemical forms. Elemental mercury (Hg^0^) emitted to the atmosphere is converted to soluble forms, deposited into soil and water, and methylated to methylmercury (MeHg). Fish and dental amalgam are two major sources of human exposure to organic (MeHg) and inorganic Hg, respectively. The exposure from dental amalgam occurs mainly by inhalation of Hg^0^ evaporating from the fillings [World Health Organization (WHO) 1991].

The central nervous system and the kidney are the primary target organs for mercury toxicity (Agency for Toxic Substances and Disease Registry 1999; Clarkson and Magos 2006; WHO 2003). The exposure and body burden of mercury can be estimated by monitoring of mercury in hair (organic Hg), blood (both organic and inorganic Hg), and urine (mainly inorganic Hg). For inorganic Hg, the urinary mercury level (U-Hg) is widely used for screening (Barregard 1993). It is influenced by the mercury concentration in the kidneys, which is higher than in most other organs.

An early effect of mercury vapor exposure is the effect on the renal proximal tubules. Low-molecular-weight proteins such as alpha-1-microglobulin (A1M) are reabsorbed slightly less effectively, and lysosomal enzymes, such as *N*-acetyl-β-d-glucosaminidase (NAG), are excreted in increased amounts. Enzymuria has been reported at occupational exposure to U-Hg of about 10–20 μg/g creatinine (Ellingsen et al. 2000). In children, however, a recent European study showed a positive association between U-Hg and urinary NAG at very low levels (< 1 μg/g creatinine) of U-Hg (de Burbure et al. 2006). Renal effects of toxic heavy metals have also be shown on brush-border enzymes, like γ-glutamyl transpeptidase (γ-GT) (Gotelli et al. 1985).

High occupational mercury exposure has been shown to increase the excretion of albumin, but mainly at U-Hg levels ≥ 100 μg/g creatinine. At lower exposure levels, albumin excretion has generally not been increased (Barregard et al. 1997; Buchet et al. 1980; Roels et al. 1985). In rare cases, high mercury exposure may cause immune-complex mediated glomerulonephritis and nephrotic syndrome (Enestrom and Hultman 1995; Tubbs et al. 1982), and animal models have been developed for mercury-induced autoimmunity (Nielsen and Hultman 2002; Pollard et al. 2005). In children too, renal effects of mercury exposure occur (Counter and Buchanan 2004). In addition a condition called acrodynia, or “pink disease,” including systemic symptoms and signs and skin rash, has been reported with inorganic mercury exposure (Dye et al. 2005; Weinstein and Bernstein 2003). This syndrome may include hypersensitivity to mercury because not all children with the same exposure were affected.

Typical U-Hg levels in adults with amalgam fillings are much lower than in those where renal effects have been shown—for example, a mean of 1.1 μg/g creatinine in a representative sample of 1,600 U.S. women 16–49 years of age in the NHANES (National Health and Nutrition Examination Study) study (Dye et al. 2005), and 1.9 μg/g creatinine in 1,100 healthy male U.S. veterans (mean age, 53 years) (Kingman et al. 1998). Similar levels are found in Canada and Central or Northern Europe (Barregard et al. 2006; Becker et al. 2003; Factor-Litvak et al. 2003). The distribution is log-normal, and U-Hg of 10–25 μg/g creatinine is found in a small fraction of the general population in the United States and Europe, for example, among heavy chewing-gum users with amalgam fillings (Barregard et al. 1995; Sallsten et al. 1996). We found no population-based study on U-Hg in U.S. children. In central Europe, however, levels are typically around 0.5 μg/g creatinine, with higher levels (1–2 μg/g creatinine) in the Mediterranean areas and coastal Canada. Dental amalgam and fish consumption are the main sources of variability (Batariova et al. 2006; de Burbure et al. 2006; Evens et al. 2001; Levy et al. 2004; Pesch et al. 2002; Trepka et al. 1997; Wilhelm et al. 2006). No systematic reviews or meta-analyses have been performed on the safety of dental amalgam.

We report here the results on markers of possible effects on the kidneys in the New England Children’s Amalgam Trial (NECAT), one of two randomized clinical trials comparing the health of children whose caries were restored using either dental amalgam or alternative materials. The first report from NECAT focused on the primary end points of neuropsychological performance. For renal markers, only an overall comparison of albumin levels in the two treatment groups was reported (Bellinger et al. 2006). No adverse neuropsycological effects of dental amalgam were found in either trial (Bellinger et al. 2006; DeRouen et al. 2006).

## Participants and Methods

A detailed description of the design of the NECAT study has been presented elsewhere (Children’s Amalgam Trial 2003), as well as details on follow-up per CONSORT (Consolidated Standards of Reporting Trials) guidelines, dental treatment, and mercury exposure (Bellinger et al. 2006). Approvals were obtained from the relevant institutional review boards.

In summary, children 6–10 years of age, with no prior or existing amalgam restorations but with two or more occlusal dental caries lesions were recruited over a 2-year period in Boston, Massachusetts, and Farmington, Maine. Baseline visits included a dental examination, blood and urine samples, anthropometric measurements, health interviews, and neuropsychological testing of the child. Exclusion criteria were clinical evidence of existing psychological, behavioral, neurologic, immunosuppressive, or renal disorders. Eligibility was confirmed for 598 children, and parental consent and child assent obtained for 534. These children were randomized to a study treatment group, stratified by geographic location (Boston vs. Farmington) and number of teeth with caries (2–4 vs. ≥ 5). Power calculations were based not on renal markers but on potential changes in IQ score. Post hoc power calculations (based on log-transformed levels) show, however, that our study had adequate power to detect an increase of NAG in the amalgam group of the size reported by de Burbure et al. (2006). For NAG and albumin we had 80% power (α = 0.05) to detect an increase in geometric means of about 20% in the amalgam group.

### Interventions and follow-up

A dispersed phase amalgam or a resin composite material (white filling) was used to restore all posterior teeth with caries at baseline and incident caries during the 5-year trial period, according to treatment group (Bellinger et al. 2006). Following standard clinical practice, however, for both groups, composite material was used to restore caries in the front teeth. Participants and dentists could not be blinded to treatment assignment, but all who collected outcome data (including interviewers) or who analyzed specimens at core laboratories were blinded to children’s treatment assignments.

Children in both groups had semiannual dental examinations. At the annual visits, neuropsychological testing and anthropometric measurements were performed and a urine sample collected (Bellinger et al. 2006). We initially attempted to collect timed overnight urine samples, but switched to spot samples mid-trial.

### Dental amalgam and mercury exposure

The number of dental restorations was highest early in the trial because of unmet treatment needs. At baseline, children had an average of 9.5 decayed tooth surfaces (range, 2–39). In subsequent years the children had on average approximately one new surface filled per year, while decidual teeth (some with fillings) were shed. In year 5, the children had 0–36 surfaces filled, with a median of four surfaces (three amalgam) in the amalgam group and five in the composite group.

**Figure 1:**
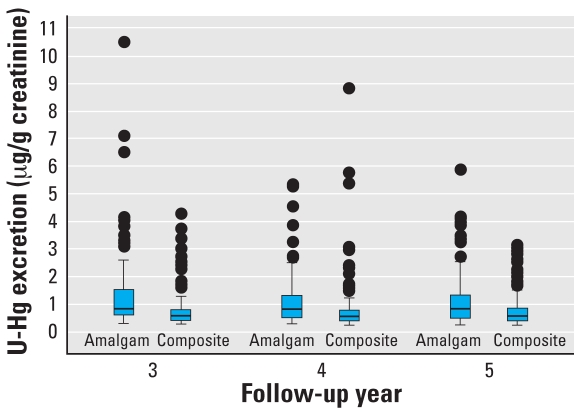
Distribution of U-Hg in children treated with amalgam or composite dental fillings in years 3–5 of the 5-year New England Children’s Amalgam Trial. The boxplots show the median (middle line), 25th and 75th percentiles (box), the extreme values (whiskers of 1.5 times the interquartile range), and the outliers (highest values).

The key measure of mercury burden was mercury in urine, corrected for creatinine (U-Hg in micrograms per gram creatinine), details on the analysis as reported by Bellinger et al. (2006). The detection limit, initially 1.5 μg/L, was reduced to 0.45 μg/L after 1 February 2000 as a result of increasing the volume of urine analyzed from each child. Nondetectable concentrations (< 0.45 μg/L) were imputed as 
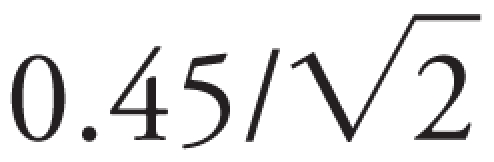
 (Hornung and Reed 1990). The mercury concentrations in the two treatment groups are shown in Figure 1. Also hair mercury levels and blood lead were measured (Bellinger et al. 2006), because methylmercury and lead could affect neuropsychological performance and kidney function.

### Renal markers

At baseline and in annual follow-up, we determined the level of γ-GT as well as a dip-stick test of proteinuria. At years 3 and 5, we added the assays of albumin, A1M, and NAG, to further assess possible mild renal effects.

γ-GT was measured in the Clinical Laboratory of the Rochester General Hospital (Rochester, NY, USA) using the Dimension system GGT Flex reagent cartridge from Dade Behring. Urinary albumin, A1M, and NAG were determined at the Sahlgrenska University Hospital. Albumin was determined by an automated nephelometric immunochemical method using reagents and calibrator from Beckman Coulter (Fullerton, CA, USA). Additional internal reference samples were used in each analytical run. The detection limit was 2.4 mg/L. The excretion of albumin was expressed in milligrams per gram creatinine.

Excretion of albumin > 30 mg/g creatinine was considered microalbuminuria (MA) (American Diabetes Association 2004). If a urine sample showed increased urinary albumin, the parents/caregivers were recommended to contact their pediatrician (or ask the NECAT trial group for help in locating a nearby clinic). While the trial was ongoing, the families were also given the option of providing a new urine sample which was reanalyzed for albumin. The criterion for taking this action was albumin > 4 mg/mmol creatinine (35 mg/g creatinine) and albumin concentration > 20 mg/L.

We determined the level of A1M by automated nephelometric immunochemical methods using reagents and calibrator from Beckman Coulter. Additional internal reference samples were used in each analytical run. The detection limit was 4 mg/L for A1M. To compare our A1M levels with most previous studies reported using antibodies from Dako (Jarup et al. 2000), we divided our A1M concentrations by 1.4 because we found that the concentrations in the two commercial antigens (calibrators) differed, with 40% higher levels using the Beckman Coulter reagents. Because only about 30% of the urine samples (with similar numbers in each treatment group) had detectable levels of A1M, we reanalyzed (2 years later) those urine samples from year 5 with sufficient volume still remaining after previous analyses, using a commercial ELISA kit for A1M from Immundiagnostik AG (Bensheim, Germany; detection limit 0.1 mg/L).

We determined total NAG with an automated photometric method based on the formation of 3-cresol purple at the reaction catalysed by NAG, using reagents and calibrator from Roche Diagnostics (Basel, Switzerland) (detection limit 0.1 U/L), and expressed in units per gram creatinine. Urinary creatinine was determined at the Swedish and the Rochester laboratories by the photometric Jaffe method (detection limit 0.1 g/L).

Urine samples were stored frozen at –20°C until analysis with median storage times of 2 months for γ-GT, 6 months for albumin and NAG, 9 months for A1M with nephelometry, and 31 months for A1M using ELISA. Owing to the range of storage times, data analysis controlled for storage time.

Twelve percent of albumin and 6% of NAG concentrations were below the level of detection, with similar numbers in each treatment group. These nondetectable concentrations were imputed as detection 
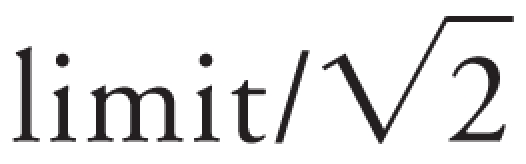
 for analysis. For A1M using nephelometry, nondetectable A1M levels were imputed as detection limit/2 for descriptive purposes only (Hornung and Reed 1990).

### Data analyses

For the assessment of possible effects on renal markers, we excluded six children with diabetes (one at baseline, five during follow-up; three in each treatment group), and six with other kidney disease (all postbaseline; three in each treatment group), as reported by parents in annual health interviews. In addition, we excluded children with increased excretion of γ-GT at baseline (seven in the amalgam group and 17 in the composite group), because they may have had a disease affecting kidney function already before dental treatment. The cutoff for γ-GT was the 95th percentile for all children at baseline (66 U/g creatinine). At each visit, there were some children who did not supply a urine sample. Additionally, some of the samples were too small to permit several analyses, so priority was given to the mercury and γ-GT analyses at the laboratory in Rochester. Consequently, the total numbers of results are lower for albumin, A1M, and NAG. Furthermore, sample sizes are lower in year 2 because of funding uncertainty and severe curtailment of data collection.

Data analyses were performed based on renal markers as continuous as well as categorical variables, the latter based on the number of high values above certain cutoff limits. For albumin we used 30 mg/g creatinine as the cutoff for MA (American Diabetes Association 2004). For urinary A1M, NAG, and γ-GT, there are no general reference limits in children. Therefore, we used the 95th percentiles in the composite group for years 1–5 (γ-GT), year 5 (A1M with ELISA), or years 3–5 (the other markers). When the renal markers were analyzed as continuous variables, levels were log-transformed.

Three outcome times were considered: *a*) year 1 for γ-GT (first visit after placement of dental fillings), *b*) years 3–5 for all markers except A1M with ELISA, and *c*) year 5 (end of the trial) for all markers. Furthermore, three predictors were used for each outcome: treatment group, number of amalgam and composite fillings (two separate variables), and U-Hg level (years 3–5 only, because a less sensitive limit of detection was used earlier in the trial).

We performed analysis of covariance (ANCOVA) (for the continuous outcomes) and logistic regression (for the categorial outcomes) for all three predictors. We used repeated-measures models with compound symmetric variance structure for the outcomes over years 3–5. All models controlled for randomization stratum, age, sex, race (non-Hispanic white, non-Hispanic black, Hispanic, other), socioeconomic status (Green 1970), baseline hair mercury level, baseline blood lead level (BPb), lean body mass, type of specimen (overnight vs. spot daytime urine sample), urinary creatinine concentration (as a surrogate for urinary flow rate), storage time, and baseline γ-GT (for γ-GT models only). In the models with U-Hg as predictor, we also tested a term for an interaction between BPb and U-Hg.

## Results

Children in the two treatment groups were similar in terms of most baseline characteristics (Table 1). The numbers of girls and boys were comparable in the amalgam group, but girls outnumbered boys in the composite group. Participants were primarily non-Hispanic white (62%), with non-Hispanic blacks comprising 19% of the sample. Baseline hair mercury and BPb were similar.

**Figure 2:**
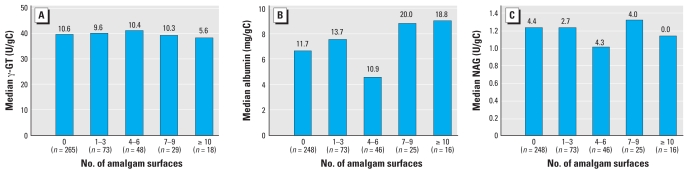
Urinary excretion of (*A*) γ-GT, (*B*) albumin, and (*C*) NAG by number of amalgam fillings in year 5 of the New England Children’s Amalgam Trial. Sample sizes are slightly higher for γ-GT because priority was given to this assay. Samples of sufficient volume were also analyzed for albumin and NAG. Numbers above the bars are percentages of (*A*) high γ-GT, (*B*) MA, and (*C*) high NAG.

The distributions for renal markers stratified by year and treatment group are shown in Table 2. The differences were small and none of them statistically significant for γ-GT, albumin, or NAG (*p* > 0.3 for ANCOVA models). Figure 2 shows median γ-GT, albumin, and NAG stratified by number of amalgam fillings at year 5. There were no significant effects of number of fillings, nor U-Hg level in ANCOVA models (*p* > 0.2 for all), except for a decrease of γ-GT in years 3–5 with increasing number of composite fillings. Models including also an interaction between BPb and U-Hg showed no such interaction for γ-GT or NAG. In the model for albumin, the interaction term U-Hg*BPb was positive and statistically significant (*p* = 0.04 for year 3–5 and *p* = 0.008 for year 5). The model indicated a 34% increase of urinary albumin at U-Hg 1.5 μg/g creatinine and BPb 4 μg/dL (90th percentiles) compared with children with median U-Hg and BPb levels (0.52 μg/g creatinine and 2 μg/dL) in year 5. For A1M, only 29% of the samples were above the detection limit (29% and 30% in the amalgam and composite groups, respectively) using nephelometry, making a treatment group comparison meaningless. With the ELISA method, the low concentrations could be quantified, but the levels had now decreased substantially after more than two years of storage. However, no significant treatment group difference was observed (*p* > 0.4 for all models).

In Table 3 we present the number of high values by treatment group and year. The overall differences between groups were not statistically significant for γ-GT, NAG, or A1M in logistic regression models. Exposure–response analyses also showed no effects of number of fillings or U-Hg level on these markers except a moderately significant decrease in the prevalence of high γ-GT with increasing numbers of both amalgam and composite fillings (*p* = 0.02 and 0.03, respectively).

However, the prevalence of urinary albumin > 30 mg/g creatinine (here called MA even if some of them had albuminuria) in year 3 or year 5 was higher in the amalgam group than in the composite group [repeated-measures logistic regression: odds ratio (OR) = 1.8; 95% confidence interval (CI), 1.1–2.9; *p* = 0.03]. The tendency was similar in year 3 and year 5 (OR = 1.8 in both cases). Crude ORs were slightly lower than those in the adjusted models (e.g., OR = 1.6; 95% CI, 0.98–2.5; *p* = 0.06) for MA in year 3–5. There was no significant increase in MA with increasing numbers of amalgam fillings (*p* = 0.30) or U-Hg excretion (*p* = 0.71). However, when an interaction term BPb*U-Hg was included in the model with U-Hg as predictor, it was statistically significant in year 5 (*p* = 0.02) and nearly so in years 3–5 (*p* = 0.07).

There were 48 occasions of MA in 38 children in the amalgam group at the 3-year and/or 5-year visits, versus 33 occasions in 31 children in the composite group. Ten children in the amalgam group (0–12 amalgam surfaces; median, 3) had MA on both visits, but only two in the composite group (*p* = 0.04; Fisher’s exact test). There was no significant interaction between treatment group and sex (*p* = 0.27). Therefore, the fact that the OR for MA was significantly increased only in boys should be considered hypothesis generating only. When urine samples with albumin > 30 mg/g creatinine but < 20 mg/L and creatinine < 0.3 g/L (i.e., samples with relatively low creatinine where the classification of MA could be considered less clear) were excluded, the results were similar (34 children with MA in the amalgam group and 9 of them on both visits, vs. 28 in the composites group and 1 of them on both visits). Twelve of the children had urinary albumin > 200 mg/g creatinine (two in year 3 only, nine in year 5 only, and one at both visits). Eight of them belonged to the amalgam group (including the one at both visits) and four to the composite group.

The exclusion of children with diabetes, kidney disease, or high γ-GT at baseline was done *a priori*, without knowledge of their albumin levels. Nevertheless, in view of the results reported above, we examined also the albumin excretion of these children. As could be expected, MA was more common in these children; in 36 children excluded *a priori*, albumin excretion was measured in 32 children, and 7 of them had MA in year 3 and/or year 5.

## Discussion

In adults, an early effect of inorganic mercury is on the renal proximal tubules. In the present study we found no indications of tubular toxicity. We used three different tubular bio-markers; a brush border enzyme (γ-GT), a lysosomal enzyme (NAG), and a low-molecular-weight protein (A1M). NAG is a commonly used biomarker in studies of tubular effects of inorganic mercury (Ellingsen et al. 2000). A recent cross-sectional study in European children found, surprisingly, a positive association between the excretion of NAG and mercury at U-Hg levels as low as in the present study (de Burbure et al. 2006). The long-term clinical significance of increased levels of NAG and A1M is unclear, but these two markers are increased and have a prognostic value in clinical renal disease (Bazzi et al. 2002; Holdt-Lehmann et al. 2000). Post hoc power calculations show that our study had adequate power to detect an increase of NAG in the amalgam group of the size reported by de Burbure et al. (2006).

Our results, indicating no effects of low-level mercury from dental amalgam on renal tubular function in children, do not contradict previous observational studies in adults showing increased urinary NAG, because the occupationally exposed subjects in those studies had U-Hg levels 10–20 times higher (Ellingsen et al. 2000). However, the aforementioned cross-sectional study of children by de Burbure et al. (2006) showed an association between U-Hg and urinary NAG in children at very low U-Hg levels, after controlling for BPb and urinary cadmium. Although a randomized controlled trial is designed to be unaffected by uncontrolled confounding factors that often constitute a problem in observational studies, the present study did not take into account cadmium and selenium status, which may influence the renal tubular toxicity of mercury (de Burbure et al. 2006; Ellingsen et al. 2000), and should be included in future studies. We found no interaction between U-Hg and BPb for the renal tubular markers. Few studies in children used γ-GT, but this marker was increased in a study of infants exposed to phenylmercury absorbed from diapers (Gotelli et al. 1985). However, mercury exposure was considerably higher than in our study.

The only finding in the present study that suggested a possible adverse effect of dental amalgam was the increased occurrence of MA. The point estimate implied an almost 2-fold risk (prevalence OR), although the lower bound of the 95% CI was 1.1. This finding was strengthened by the fact that 10 of 12 cases with persistent MA were found in the amalgam group.

High industrial exposure to inorganic mercury increases the prevalence of albuminuria in adults (Buchet et al. 1980). Moreover, high exposure may cause membranous glomerulonephritis with nephrotic syndrome, in industry (Tubbs et al. 1982) as well as in the general population (Oliveira et al. 1987). Within groups with the same exposure, only some people are affected, and increased susceptibility may be associated with *HLA* phenotype (Enestrom and Hultman 1995). This is also true for rare mercury hypersensitivity reactions in children (Clarkson and Magos 2006; Counter and Buchanan 2004; Weinstein and Bernstein 2003). These effects, however, have never been shown at low-level exposure. Animal models indicate that the mechanisms behind mercury-induced auto-immunity are complex, and genetic factors, especially *MHC* genes, may be important (Nielsen and Hultman 2002; Pollard et al. 2005; Tubbs et al. 1982). In view of previous knowledge, our finding of MA in children treated with dental amalgam may therefore represent a causal association. If so, our findings indicate that children with high BPb may be more sensitive than others.

However, chance is clearly an alternative explanation for a higher occurrence of MA in the present study. We excluded children with diabetes or known kidney disease, but temporary albuminuria is relatively common and may be caused by strenuous physical exercise, urinary tract infections, or other conditions with fever, or it may represent so-called orthostatic proteinuria (Hogg et al. 2000). The NHANES study reported the prevalence of MA in U.S. children and adolescents (6–19 years of age) to be 6.2% in males and 13.4% in females, when individuals with diabetes or other relevant diseases had been excluded (Jones et al. 2002). This is very similar to the prevalence in the composite group of 9–10% (boys and girls combined), whereas the prevalence in the amalgam group was somewhat higher. The higher prevalence in girls than in boys is in accordance with previous knowledge (Jones et al. 2002). The median albumin levels were in agreement with those reported for U.S. or European children (Jones et al. 2002; Lehrnbecher et al. 1998). Our samples were stored frozen for 6 months on average, which probably led to some decrease of initial levels. The samples from the NHANES study, also stored frozen, showed a similar median albumin level as the NECAT study.

We searched for two kinds of renal effects: glomerular and tubular. We tested effects on mean levels as well as on increased prevalence in high levels. It is possible that the difference in one (MA) of these four major outcomes is just a random finding attributed to multiple testing. At the same time as the NECAT study another controlled clinical trial, conducted in Portugal with a nearly identical design, was published (DeRouen et al. 2006). No increase in the occurrence of MA has been found in the Portuguese study (B. Leroux, personal communication). Unless there is a genetic difference in susceptibility between children in the Mediterranean region and most children in the Northeast United States, an absence of increased MA in the Casa Pia study (B. Leroux, personal communication) makes a causal association in the present study less likely.

Although we found 10 children in the amalgam group with MA in both years 3 and 5, we do not know whether these children will have persistent MA. A more extensive clinical investigation of these children would have been valuable. However, the protocol of this trial did not permit us to investigate further. Therefore, the clinical implications are unclear, should the increase of MA in the amalgam group reflect a causal association. A reversible MA may be harmless, such as after many viral infections or strenuous exercise (Hogg et al. 2000). MA reflecting a long-lasting effect on glomerular integrity would, however, be a serious side effect even if only a small fraction of children were affected. A study specifically focused on the possible association between dental amalgam and MA in children would be desirable. Another randomized controlled study would be optimal but may require very large groups. An alternative would be case–referent or case–crossover studies in surroundings where substantial fractions of children have and have not had dental amalgam fillings.

The U-Hg levels of the children assigned to the amalgam group were similar to or slightly higher than those in U.S. adults with a similar number of dental amalgam surfaces (Dye et al. 2005; Kingman et al. 1998). Given that the children enrolled had a considerably higher prevalence of caries than in other U.S. children of comparable age, the children assigned to the amalgam group are likely to have experienced higher exposure to mercury from amalgam than the average U.S. child. Nevertheless, the findings should be relevant for most U.S. children. A study of 100 low-income, inner-city children from New York City showed a mean U-Hg of 1.1 μg/L (Ozuah et al. 2003).

This trial has several strengths: First, we recruited children in whom no dental amalgam restorations had ever been placed and assigned them randomly to treatment group. This experimental design contributed to an equivalence of treatment groups, at baseline, on measured and unmeasured factors that could create confounding or effect modification. Second, we recruited children with many dental caries, providing a setting in which the study hypotheses could be adequately tested. Third, the design included a long term follow-up with measurements of renal markers on several occasions. Fourth, loss to follow-up was remarkably low for a U.S. study, with 5-year renal outcome data for 81% of the children enrolled.

There are, however, also several limitations. First, because the main focus was on neuropsychological function, the baseline ascertainment of renal function was limited: interviews on possible renal disease, a dip-stick, and measurement of urinary γ-GT. This screening should, however, detect most of the children with possible kidney disorders at baseline. Second, the assessment of one of the three tubular markers, A1M, was not optimal because of a high detection limit (nephelometry) or long storage time (ELISA). Third, we used spot samples adjusted for creatinine, although 24-hr excretion will have somewhat lower intraindividual variability. Fourth, although this trial was adequately powered to detect a small treatment-group difference in averages, it has low power to catch an effect that occurs only in a small susceptible fraction of children. Fifth, a follow-up period > 5 years might be needed to appreciate potential subtle toxic effects of exposure to mercury from dental amalgam, such as on renal proximal tubules.

In summary, the present randomized clinical trial showed no effect of amalgam on renal tubular function. There was, however, an increased prevalence of MA in children treated with dental amalgam. This may reflect a causal association or it may be a chance finding. This issue should be examined further.

## Figures and Tables

**Table 1:** Baseline characteristics of NECAT participants (*n* = 534^a^).

Characteristic	Amalgam group (*n* = 267)	Composite group (*n* = 267)
Site [*n* (%)]		
Boston, Massachusetts	144 (53.9)	147 (55.1)
Farmington, Maine	123 (46.1)	120 (44.9)
No. of carious surfaces [mean ± SD (range)]	9.8 ± 6.9 (2–39)	9.3 ± 6.2 (2–36)
Age [mean ± SD (range)]	7.9 ± 1.3 (5.9–11.4)	7.9 ± 1.4 (5.9–11.5)
Sex [*n* (%)]		
Female	131 (49.1)	156 (58.4)
Male	136 (50.9)	111 (41.6)
Race [*n* (%)]^b^		
Non-Hispanic white	165 (64.0)	158 (60.3)
Non-Hispanic black	49 (19.0)	49 (18.7)
Hispanic	15 (5.8)	23 (8.8)
Other	29 (11.2)	32 (12.2)
Household income [*n* (%)]		
≤ $20,000	74 (29.2)	86 (33.1)
$20,001–$40,000	113 (44.7)	109 (41.9)
> $40,000	66 (26.1)	65 (25.0)
Education of primary caretaker [*n* (%)]		
< High school	34 (13.2)	38 (14.6)
High school graduate	197 (76.4)	194 (74.3)
College graduate	18 (7.9)	17 (6.5)
Postcollege degree	9 (3.5)	12 (4.6)
Hair mercury [μg/g (mean ± SD)]	0.4 ± 0.5	0.4 ± 0.5
BPb [μg/dL (mean ± SD)]	2.4 ± 1.9	2.3 ± 1.5

a The number of trial participants includes those who later withdrew (85 of 534, 42 in the amalgam group and 43 in the composite group). For race, data were available for 520 participants; for income, 513; for education, 519.

b Race was self-reported by the parents of the children. The other category included individuals who identified themselves as Asian, Pacific Islander, Native American, biracial, or other, which they were asked to specify.

**Table 2:** Summary of the results for renal markers at baseline and follow-up in 490^a^ children [median (no.) and range].

	Baseline	Year 1	Year 2	Year 3	Year 4	Year 5^b^
γ-GT (U/g creatinine)						
Amalgam group	19.5 (238)	19.4 (186)	26.4 (126)	31.9 (185)	36.5 (192)	39.3 (204)
	2.1–66	1.5–73	< 1–73	1.1–100	< 1–146	3.6–125
Composite group	17.4 (223)	19.7 (182)	24.5 (139)	34.5 (180)	36.9 (173)	40.2 (198)
	2.0–62	1.0–89	< 1–124	0.9–247	1.5–132	2.6–143
Albumin^c^ (mg/g creatinine)						
Amalgam group				6.8 (135)		6.0 (193)
				< DL–773		< DL–771
Composite group				7.9 (148)		6.5 (186)
				< DL–208		< DL–687
NAG^c^ (U/g creatinine)						
Amalgam group				1.4 (135)		1.2 (193)
				< DL–4.7		< DL–3.7
Composite group				1.4 (148)		1.2 (186)
				< DL–4.8		< DL–7.8
A1M^c^ (mg/g creatinine)						
Amalgam group				< DL (135)		< DL (193)^d^
				< DL–29		< DL–29
Composite group				< DL (148)		< DL (186)^d^
				< DL–21		< DL–29

DL, detection limit.

a Of the 534 children, 36 were excluded from analysis because of parent-reported diabetes (*n* = 6), other renal disease (*n* = 6), or high baseline γ-GT (*n* = 24). An additional 8 children never provided urine samples throughout the trial.

b In ANCOVA models for treatment group, controlling for randomization stratum, age, sex, race, socioeconomic status, baseline hair mercury, baseline BPb, lean body mass, time of specimen (overnight vs. daytime), creatinine concentration, storage time, and baseline γ-GT (for γ-GT models only), *p* = 0.86 for γ-GT, *p* = 0.46 for albumin, *p* = 0.95 for NAG, and *p* = 0.79 for A1M.

c Samples below the detectable concentration were imputed as detection 
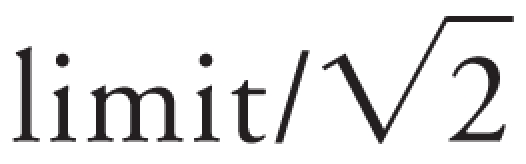
(for albumin and NAG) or detection limit/2 (for A1M) for computation of the medians and maximums.

d Reanalysis of A1M from year 5 using ELISA showed a median A1M of 0.66 (range, 0.06–5.0) mg/g creatinine (*n* = 90) in the amalgam group and 0.55 (range, 0.07–7.4) mg/g creatinine (*n* = 97) in the composite group, *p* = 0.80.

**Table 3:** Prevalence of high values^a^ for renal markers at follow-up in 458 children [no./total sample (%)].^b^

	Year 1	Year 2	Year 3	Year 4	Year 5^c^
γ-GT (U/g creatinine)					
Amalgam group	2/186 (1.1)	1/126 (0.8)	13/185 (7.0)	16/192 (8.3)	20/204 (9.8)
Composite group	2/182 (1.1)	6/139 (4.3)	13/180 (7.2)	13/173 (7.5)	20/198 (10)
Albumin^d^ (mg/g creatinine)					
Amalgam group			18/135 (13)		30/193 (16)
Composite group			15/148 (9.5)		18/186 (9.7)
NAG^d^ (U/g creatinine)					
Amalgam group			5/135 (3.7)		5/193 (2.6)
Composite group			8/148 (5.4)		8/186 (4.3)
A1M^d^ (mg/g creatinine)					
Amalgam group			5/135 (3.7)		5/193 (2.6)^e^
Composite group			13/148 (8.8)		3/186 (1.6)^e^

a Cutoffs were 71.9 U/g creatinine for γ-GT, 30 mg/g creatinine for albumin, 3.1 U/g creatinine for NAG, 10.5 mg/g creatinine for A1M (nephelometry), and 3.7 mg/g creatinine for A1M (ELISA). These were chosen as the 95th percentile of the composite group, except for albumin, which uses a standard cutoff.

b Of the 534 children, 36 were excluded from analysis because of parent-reported diabetes (*n* = 6), other renal disease (*n* = 6) or high baseline γ-GT (*n* = 24). Eight children never provided urine samples, and an additional 32 children never provided samples after baseline.

c In logistic regression models for treatment group, controlling for randomization stratum, age, sex, race, socioeconomic status, baseline hair mercury, baseline BPb, lean body mass, time of specimen (overnight vs. daytime), creatinine concentration, storage time, and baseline γ-GT (for γ-GT models only), *p* = 0.85 for γ-GT, *p* = 0.07 for albumin, *p* = 0.59 for NAG, and *p* = 0.89 for A1M.

d Samples below the detectable concentration were imputed as detection 
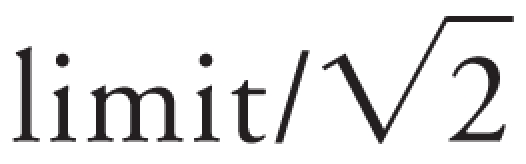
 (for albumin and NAG) or detection limit/2 (for A1M).

e Reanalysis of A1M from year 5 using ELISA showed 1/90 (1.1%) and 4/97 (4.1%) high A1M in the amalgam and composite groups, respectively, *p* = 0.11.
